# Dynamics of peripheral T cell exhaustion and monocyte subpopulations in neurocognitive impairment and brain atrophy in chronic HIV infection

**DOI:** 10.1007/s13365-024-01223-w

**Published:** 2024-06-29

**Authors:** Brooks I. Mitchell, Isabelle E. Yazel Eiser, Kalpana J. Kallianpur, Louie Mar Gangcuangco, Dominic C. Chow, Lishomwa C. Ndhlovu, Robert Paul, Cecilia M. Shikuma

**Affiliations:** 1 Hawaii Center for AIDS, John A. Burns School of Medicine, University of Hawaii at Manoa, 651 Ilalo St., Biomedical Sciences Building 231, Honolulu, HI 96813 USA; 2 Department of Tropical Medicine, John A Burns School of Medicine, University of Hawaii, Honolulu, HI USA; 3 Kamehameha Schools– Kapālama, Honolulu, HI USA; 4 Department of Medicine, John A Burns School of Medicine, University of Hawaii, Honolulu, HI USA; 5 Division of Infectious Diseases, Department of Medicine, Weill Cornell Medicine New York, New York, USA; 6 Department of Psychological Sciences, Missouri Institute of Mental Health, University of Missouri-St. Louis, St. Louis, MO USA

**Keywords:** Human immunodeficiency Virus-1 (HIV), Cognition, Brain volumes, MRI, Monocytes, T cells, PD-1, TIGIT, Immune exhaustion

## Abstract

**Background:**

HIV-associated neurocognitive disorders (HAND) is hypothesized to be a result of myeloid cell-induced neuro-inflammation in the central nervous system that may be initiated in the periphery, but the contribution of peripheral T cells in HAND pathogenesis remains poorly understood.

**Methods:**

We assessed markers of T cell activation (HLA-DR + CD38+), immunosenescence (CD57 + CD28-), and immune-exhaustion (TIM-3, PD-1 and TIGIT) as well as monocyte subsets (classical, intermediate, and non-classical) by flow cytometry in peripheral blood derived from individuals with HIV on long-term stable anti-retroviral therapy (ART). Additionally, normalized neuropsychological (NP) composite test z-scores were obtained and regional brain volumes were assessed by magnetic resonance imaging (MRI). Relationships between proportions of immune phenotypes (of T-cells and monocytes), NP z-scores, and brain volumes were analyzed using Pearson correlations and multiple linear regression models.

**Results:**

Of *N* = 51 participants, 84.3% were male, 86.3% had undetectable HIV RNA < 50 copies/ml, median age was 52 [47, 57] years and median CD4 T cell count was 479 [376, 717] cells/uL. Higher CD4 T cells expressing PD-1 + and/or TIM-3 + were associated with lower executive function and working memory and higher CD8 T cells expressing PD-1^+^ and/or TIM-3^+^ were associated with reduced brain volumes in multiple regions (putamen, nucleus accumbens, cerebellar cortex, and subcortical gray matter). Furthermore, higher single or dual frequencies of PD-1 + and TIM-3 + expressing CD4 and CD8 T-cells correlated with higher CD16 *+* monocyte numbers.

**Conclusions:**

This study reinforces evidence that T cells, particularly those with immune exhaustion phenotypes, are associated with neurocognitive impairment and brain atrophy in people living with HIV on ART. Relationships revealed between T-cell immune exhaustion and inflammatory in CD16^+^ monocytes uncover interrelated cellular processes likely involved in the immunopathogenesis of HAND.

**Supplementary Information:**

The online version contains supplementary material available at 10.1007/s13365-024-01223-w.

## Introduction

HIV-associated neurological complications including structural and functional brain alterations as well as neurocognitive disorders present a serious concern for people living with HIV (PLWH). Structural magnetic resonance imaging (sMRI) studies have indicated widespread atrophy in a number of brain regions as well as cerebellar and total subcortical gray matter volume differences indicating brain abnormalities in HIV-positive individuals(Kallianpur et al. 2013; Ma et al. 2021; Nir et al. 2019) Lower CD4 + T cell levels have been previously associated with smaller hippocampal and thalamic volumes in those on ART and lower putamen volumes in those not on antiretroviral therapy (ART). Brain atrophy in both gray and white matter regions is associated with cognitive decline and can result despite long-term stable use of ART(Nir et al. 2021/01; Nir et al. 2019). HIV-associated neurocognitive disorder, also known as HAND has a prevalence that is estimated to be as high as 30–50% and encompasses a spectrum of neurocognitive disease in PLWH(Heaton et al. 2011; Tozzi et al. 2007). Neurocognitive impairment seen in individuals treated early with ART is predominantly mild and characterized by decreased information processing speed and memory deficits, however gray matter volume has been shown to accurately discriminate between HAND patients, PLWH but no neurocognitive impairment, and control(Schantell et al. 2021; Tozzi et al. 2007). Despite mild symptomatology, HAND impacts daily functioning and is associated with increased morbidity and mortality (Benedict et al. 2000; Morgan et al. 2012). To date, there are no clinically proven therapies for HAND for individuals already on stable, virally suppressive ART.

In autopsy studies of people with HIV, histological evaluation has demonstrated a high degree of residual chronic inflammation in brain tissue and increased presence of macrophages and microglial cells despite ART(Anthony et al. 2005). Associations of clinical measures of HAND to blood and cerebrospinal fluid (CSF) markers of monocyte/macrophage activation (e.g., neopterin, sCD163 and sCD14) suggest the importance of the myeloid compartment in the pathogenesis of HAND (Agsalda-Garcia et al. 2017; Brown 2015; Kusao et al. 2012; Ndhlovu et al. 2015; Rappaport and Volsky 2015; Williams et al. 2014). Other biomarkers such as HIV DNA, autoantibodies, and inflammatory cytokines present in the CSF as well as alterations in T-cell abundance and CD4/CD8 T-cell ratios in the peripheral blood have been associated with altered neurocognitive functioning in PLWH but are still unable to be used for diagnostic purposes(Abassi et al. 2017; Gougeon et al. 2017; Koffler et al. 2018; Vassallo et al. 2016). These biomarker associations, however, fail to account for the association of HAND to alterations in T cell specific immunological markers. T cell markers associated with HAND include low nadir CD4 T cell counts, low CD4/CD8 T cell ratios, increased frequencies of activated CD4 and CD8 T cells measured both in the periphery and in the CSF, higher levels of the T cell chemo-attractant CXCL10, and presence of CD8 T cells expressing IFN-γ in the CSF (Ellis et al. 2011; Mehla et al. 2012; Schrier et al. 2015; Vassallo et al. 2016). Furthermore, recent studies have identified the prevalence and persistence of HIV in freshly isolated CSF derived CD4 T cells in ART suppressed individuals with HIV (Farhadian et al. 2022). These T cell specific associations suggest that T cells may play a larger role in the pathogenesis of HAND than is currently recognized.

T cell exhaustion, is characterized by the upregulation of negative checkpoint receptors (NCRs) and has recently been of major interest in HIV cure research as it is linked to the persistence of the latent HIV reservoir (Chew et al. 2016; Fromentin et al. 2016). PD-1 is an NCR that is upregulated in chronic viral infections and is associated with suppression of T cells in parenchymal tissues as well as T cell exhaustion (Manenti et al. 2022; Said et al. 2010). Higher levels of PD-1 expression on CD4 T cells is associated with worsening neurocognitive impairment in people living with HIV (Grauer et al. 2015; Said et al. 2010). Furthermore, PD-1 expression has been found to be upregulated on T cells in other neuroinflammatory conditions and resulting in a reduction of Th1 cells; however, the exact mechanisms associated with CNS injury are still unclear (Manenti et al. 2022). Less is known about the impact of NCRs and T cell exhaustion on non-infectious chronic complications of HIV including HAND. In this study, we sought to assess the relationship of primary (PD-1) and secondary tier (TIGIT and TIM-3) NCRs on T cells to measures of brain integrity (NP impairment and to regional brain volumes) in PLWH. We report that T cell immune exhaustion is associated with worse cognition and decreased brain volumes.

## Methods

### Study participants

This retrospective study was conducted utilizing entry data and specimens from the Hawaii Aging with HIV Cohort-Cardiovascular Disease (HAHC-CVD) study, a 5-year natural history study investigating the role of chronic HIV infection in the development of cardiovascular disease (CVD) among PLWH on suppressive ART. Inclusion criteria required documented HIV-positive status, age ≥ 40 years, and use of combination ART ≥ 3 months. Patients who were institutionalized or with active malignancy, infection or AIDS-defining illness at the time of enrollment were excluded. Due to the use of MRI in this study, the following exclusion criteria were employed: (i) uncontrolled major affective disorder; (ii) active psychosis; (iii) loss of consciousness > 5 min; (iv) pregnancy or breast feeding; (v) factors that would preclude MRI (e.g. claustrophobia); and (vi) any past or present condition (central nervous system infection, brain trauma, stroke, substance abuse, *ect.*) deemed by the evaluating physician to introduce confounding variables. Extensive HIV immunologic assessments were available from this cohort and further details have been previously described (Shikuma et al. 2012). A subset of this cohort underwent NP testing and brain MRI. IRB approval was obtained from the University of Hawaii Human Studies Program (CHS #16,476), and all participants provided written informed consent prior to enrollment into the HAHC-CVD study. The study was conducted in accordance with the Declaration of Helsinki and adhered to the guidelines of the University of Hawaii IRB. At the time of entry into the cohort, all participants gave written informed consent to allow use of clinical data and banked specimens for future studies related to HIV and its complications. All banked specimens and data collected from participants were anonymized and de-identified prior to analysis.

### Neuropsychological testing

Participants completed a comprehensive 80-minute NP testing battery at the time of study entry. NP testing was conducted by trained psychometrists at the University of Hawaii Clinics at Kakaako with quality assurance of testing procedures provided by a neuropsychologist (Robert H. Paul Ph.D.). Participants were tested in a quiet room to minimize distractions and provided breaks as needed. The NP battery involved a series of tests that were selected to assess the function of specific cognitive domains (learning and memory, psychomotor speed, working memory, and executive function) known to be sensitive to HIV-related cognitive dysfunction as shown in Table 1. As previously described, raw NP test scores were z-transformed by use of age, gender and education-adjusted normative data. Composite, domain specific z-scores (psychomotor speed [NPZpm]; learning and memory [NPZlrn-mem]; executive functioning [NPZef]; working memory [NPZwm]) were derived by averaging z-scores of the appropriate individual tests. A global z score (NPZ-global) was the arithmetic mean of z-scores on 14 NP tests(Chai et al. 2018; Kallianpur et al. 2016b; Umaki et al. 2013). Normative data were adapted from the Multicenter AIDS Cohort Study, which included PLWH and HIV-seronegative individuals (Valcour and Paul 2006). Diagnosis of HAND were not made in this study.

**Table 1:** Assessment tests for global and domain-specific NP performance.

Neuropsychological Domain	Assessment Measurements
Global	WAIS-IV Digit Symbol, Trail-Making Test Part A, Trail-Making Test Part B, Grooved Pegboard (Dominant, Non-Dominant Hand), California Computerized Assessment Package (CalCAP)(Choice RT, Sequential RT), Timed Gait, California Verbal Learning Test (CVLT-II) (Interference, Long-Delay Free Recall), Brief Visual Spatial Memory Test (BVMT-R) (Delayed Recall), D-KEFS Color-Word Interference Test (Stroop), Verbal Fluency (FAS), Category Fluency (Action Naming Test)
Learning and Memory	CVLT-II (Total Recall, Long Delay Free Recall), BVMT-R (Delayed Recall)
Executive Function	FAS, Stroop, Trail-Making Test Part B, Action Naming Test
Working Memory	WAIS-IV Digit Span, CVLT-II (Interference Trial), WAIS-IV Letter Number Sequencing
Psychomotor Speed	WAIS-IV Digit Symbol, Trail-Making Test Part A, Trail-Making Test Part B, Grooved Pegboard (Dominant, Non-Dominant Hand)

### Neuroimaging

Neuroimaging was performed as previously described (Kallianpur et al. 2016a). Briefly, MRIs were conducted on a 3.0-Tesla Philips Medical Systems Achieva scanner equipped with an 8-channel head coil (InVision Imaging, Honolulu). For each participant, a high-resolution anatomical volume was acquired with a sagittal T1-weighted 3D turbo field echo (T1W 3D TFE) sequence (echo time TE/repetition time TR = 3.2 ms/6.9 ms; flip angle 8°; slice thickness 1.2 mm with no gap; in-plane resolution 1.0 mm^2^; field of view 256 × 256 mm^2^). To obtain regional brain volumes, structural MRI data were processed with FreeSurfer (version 5.0, https://surfer.nmr.mgh.harvard.edu/) in a procedure that includes skull-stripping (Segonne et al. 2004), intensity normalization (Sled et al. 1998), Talairach transformation, subcortical white matter and deep gray matter segmentation (Fischl 2004; Fischl et al. 2002), and cortical gray/white matter boundary and pial surface reconstruction (Dale et al. 1999). Quality assurance of processed data was performed visually, and cortical surfaces and subcortical segmentations were checked prior to statistical analysis. FreeSurfer’s estimate of intracranial volume (ICV) is a reliable measure for regional brain volume normalization (Buckner et al. 2004).

### PBMC isolation and flow cytometry assay

PBMC isolation and flow cytometry assays were performed as previously reported (Chew et al. 2016). In brief, whole blood was drawn into EDTA tubes at the time of study entry and cells were processed for peripheral blood mononuclear cells (PBMCs) isolation within one hour of collection and cryopreserved. Banked cryopreserved PBMCs were thawed and washed with warmed AIM-V liquid media (Invitrogen). For detection of T cell activation, senescence, and exhaustion thawed cryopreserved PBMCs were stained with AARD to exclude nonviable cells and for surface expression of CD3, CD28, CD4, CD38, HLA-DR, CD8, CD57, PD-1, TIM-3, and TIGIT. Data were acquired on a custom 4-laser BD LSRFortessa Cell Analyzer and all compensation and gating analyses were performed in FlowJo analytical software to quantitate CD4 and CD8 T cells expressing activation (CD38 and HLADR), senescence (CD28 and CD57), and exhaustion (PD-1, TIM-3, TIGIT) markers. Data are expressed as the percentage of T cells expressing these markers. For monocyte immunophenotyping, PBMCs were surface-stained with CD3, CD14, CD16, CD56, CD19, CD20, HLA-DR antibodies, and with Live/Dead fixable yellow dead cell stain. Data were acquired on a custom 4-laser BD LSRFortessa Cell Analyzer and all compensation and gating analyses were performed in FlowJo analytical software (gating strategy previously published(Shikuma et al. 2014). Percentages of classical, intermediate, and non-classical monocyte subsets were determined based on CD14 and CD16 staining and absolute numbers of each subset were calculated from WBC and monocyte percent obtained from available clinical CBC performed on each participant from the same blood draw.

### Statistical analysis

Demographic, clinical, and immunologic information were summarized by median (interquartile range) values for continuous variables and frequency (percentage) for categorical variables. Correlation between immunologic parameters, neurocognitive receptor domains, and brain volumes were assessed by Pearson’s correlation. Multivariable linear regression analyses were used to assess the association of exhausted T cells with NP z-scores and brain volumes. Statistical analyses were performed using IBM SPSS version 22 software and statistical significance was set at a *p*-value < 0.05. Continuous variables with skewed distributions were log-10 transformed prior to analyses.

## Results

### Baseline characteristics of participants

A total of 51 participants enrolled into the HAHC-CVD cohort had available NP performance testing and brain imaging and were included in this analysis. The demographic, clinical, and immunologic characteristics of the participants are summarized in Table 2. The majority of participants were male with a median age of 51 years. Participants were mostly of Caucasian ethnicity and had a median current CD4 T cell count of 479 [376, 717] cells/mm^3^. The median nadir CD4 count was much lower at 92.0 [33.0, 198.25] cells/mm^3^. Undetectable plasma HIV viral load, defined as HIV RNA 50 copies/ml, was noted in 86.3% of the participants. Overall NP performance in our population was within normal limits, with slightly worse performance on learning and memory subdomains.

**Table 2:** Baseline characteristics of study participants.

**Demographic and Clinical Parameters**	
Characteristic	*n* = 51
Age, years	52 [47, 57]
Male, n (%)	43 (84.3%)
Race, n (%) Caucasian	30 (58.8%)
Duration of education, years	14 [12, 16]
Duration since HIV diagnosis, years	17 [11, 20]
Duration since ART initiation, years	14 [8, 17]
HIV RNA < 50 copies/ml, n (%)	44 (86.3%)
Neuropsychological performance, z-score	
Global-14 Learning and memory Executive function Working memory Psychomotor speed	-0.05 [-0.48, 0.32] -0.66 [-1.26, 0.55] 0.27 [-0.38, 1.00] -0.04 [-0.45, 0.40] 0.27 [-0.16, 0.72]
Structural Brain Volumes, mm^3^	
Thalamus Caudate Putamen Pallidum Hippocampus Amygdala Nucleus Accumbens Cerebellar White Matter Cerebellar Gray Matter Cerebral White matter Subcortical Gray Matter Intracranial Volume	13.0 [12.2,15.0] 7.3 [5.9, 9.4] 11.4 [10.2, 12.5] 3.3 [2.1, 5.9] 7.9 [5.2, 8.2] 3.8 [1.3, 5.8] 1.3 [0.8, 2.0] 30.9 [28.2, 32.5] 87.4 [82.3, 88.3] 43.1 [42.2, 44.1] 164534.5 [159634.1, 170754.2] 1451.2 [1145.2, 1562.8]
**Immunological Parameters**	
Nadir CD4^+^ T cells, cells/µL	92.0 [33.0, 198.25]
CD4^+^ T cells, cells/µL	496.50 [374.5, 554.5]
Senescent CD4^+^ T cells (CD57^+^CD28^−^), %	2.30 [0.85, 9.81]
Negative checkpoint expression on CD4^+^T cells, %	
PD-1^+^ TIGIT^+^ TIM-3^+^ PD-1^+^TIGIT^+^ PD-1^+^TIM-3^+^ TIGIT^+^TIM-3^+^	41.2 [29.4, 54.1] 22.7 [16.4, 30.6] 16.2 [13.0, 20.4] 16.7 [11.2, 23.1] 7.6 [5.0, 9.4] 4.1 [3.1, 5.4]
CD8^+^ T cells, cells/µL	885.5 [533.0, 1160.0]
Activated CD8^+^ T cells (CD38^+^HLA-DR^+^), %	9.91 [6.17, 17.10]
Senescent CD8^+^ T cells (CD57^+^CD28^−^), %	18.35 [12.55, 23.78]
Negative checkpoint expression on CD8^+^T cells, %	
PD-1^+^ TIGIT^+^ TIM-3^+^ PD-1^+^TIGIT^+^ PD-1^+^TIM-3^+^ TIGIT^+^TIM-3^+^	38.7 [23.6, 50.0] 44.0 [35.4, 61.5] 24.5 [19.7, 29.2] 24.6 [16.9, 35.9] 6.5 [3.9, 8.5] 9.6 [7.5, 13.3]
Total monocytes, %	6.8 [5.2, 8.1]
Classical (CD14^++^CD16^−^) monocytes, cells/L	3.4 × 10^8^ [2.4 × 10^8^, 4.6 × 10^8^]
Classical (CD14^++^CD16^−^) monocytes, %	89.1 [85.1, 93.2]
Intermediate (CD14^++^CD16^+^) monocytes, cells/L	7.2 × 10^6^ [2.6 × 10^6^, 13.8 × 10^6^]
Intermediate (CD14^++^CD16^+^) monocytes, %	3.1 [2.4, 4.1]
Non-classical (CD14^+/low^CD16^++^) monocytes, cells/L	5.2 × 10^6^ [2.2 × 10^6^, 10.2 × 10^6^]
Non-classical (CD14^+/low^CD16^++^) monocytes, %	4.3 [3.8, 6.2]

a. Median values shown with [median Q1, median Q3] or frequency, n with (%)

### Relationship between NCR-expressing T cells and clinical parameters, T cell immune activation and senescence

CD4 and CD8 T cells were evaluated by flow-cytometry for single and double expression of three known NCRs: PD-1, TIM-3, and TIGIT (Table 2). Pearson correlations of single and double NCR-expressing CD8 and CD4 T cells with other T cell parameters (current CD4 T cell count, CD4 CD8 T cell ratio, nadir CD4 count, % activated T cells, and % senescent T cells) are shown in Table 3. Higher percentages of CD4 and CD8 T cells single positive for TIGIT or PD-1 correlated with lower current CD4 T cell counts and CD4/CD8 T cell ratios. Higher frequencies of TIGIT or PD-1 single positive CD4 T cells, but not with CD8 T cells, correlated with lower nadir CD4 T cell counts. Higher frequencies of PD-1 single positive CD4 T cells and TIM-3 single positive CD8 T cells correlated with higher frequencies of senescent (CD57 + CD28-) CD8 T cells. Higher frequencies of CD4 and CD8 T cells single positive for PD-1 and TIGIT correlated with higher frequencies of activated (CD38 + HLA-DR+) CD8 T cells. There were no correlations found between CD4 T cells single positive for TIM-3 with other T cell parameters.

**Table 3:** Pearson correlations between NCR-expressing CD4 and CD8 T cells.

	Current CD4 count	CD4 CD8 Ratio	Nadir CD4 count	Senescent CD4	Senescent CD8	Activated CD8
*Single positive*:						
CD8 + TIGIT+ CD8 + PD-1+ CD8 + TIM-3+ CD4 + TIGIT+ CD4 + PD-1+ CD4 + TIM-3+	*r*=-0.501* *p* = 0.003 *r*=-0.448* *p* = 0.009 *r*=-0.302 *p* = 0.087 *r*=-0.698** *p* < 0.0001 *r*=-0.783** *p* < 0.0001 *r* = 0.007 *p* = 0.968	*r*=-0.525* *p* = 0.002 *r*=-0.466* *p* = 0.007 *r*=-0.294 *p* = 0.102 *r*=-0.723** *p* < 0.0001 *r*=-0.685** *p* < 0.0001 *r* = 0.143 *p* = 0.436	*r*=-0.148 *p* = 0.420 *r*=-0.288 *p* = 0.109 *r*=-0.277 *p* = 0.125 *r*=-0.417* *p* = 0.018 *r*=-0.495* *p* = 0.004 *r* = 0.046 *p* = 0.801	*r* = 0.163 *p* = 0.373 *r* = 0.002 *p* = 0.993 *r* = 0.000 *p* = 0.998 *r*=-0.008 *p* = 0.966 *r* = 0.274 *p* = 0.130 *r* = 0.318 *p* = 0.076	*r* = 0.291 *p* = 0.106 *r* = 0.130 *p* = 0.477 *r* = 0.437* *p* = 0.012 *r* = 0.336 *p* = 0.060 *r* = 0.442* *p* = 0.011 *r* = 0.170 *p* = 0.353	*r* = 0.551* *p* = 0.004 *r* = 0.484* *p* = 0.012 *r* = 0.261 *p* = 0.198 *r* = 0.420* *p* = 0.033 *r* = 0.476* *p* = 0.014 *r* = 0.081 *p* = 0.695
*Dual positive*:						
CD8 + PD-1+ TIM-3+ CD8 + PD-1 + TIGIT+ CD8 + TIM-3 + TIGIT+ CD4 + PD-1+ TIM-3+ CD4 + PD-1 + TIGIT+ CD4 + TIM-3 + TIGIT+	*r*=-0.444* *p* = 0.010 *r*=-0.436* *p* = 0.011 *r*=-0.453* *p* = 0.008 *r*=-0.598** *p* < 0.0001 *r*=-0.729** *p* < 0.0001 *r*=-0.583** *p* < 0.0001	*r*=-0.383* *p* = 0.030 *r*=-0.466* *p* = 0.007 *r*=-0.379* *p* = 0.032 *r*=-0.465* *p* = 0.007 *r*=-0.683** *p* < 0.0001 *r*=-0.589** *p* < 0.0001	*r*=-0.473* *p* = 0.006 *r*=-0.199 *p* = 0.275 *r*=-0.255 *p* = 0.159 *r*=-0.329 *p* = 0.066 *r*=-0.496* *p* = 0.004 *r*=-0.291 *p* = 0.107	*r* = 0.017 *p* = 0.927 *r*=-0.072 *p* = 0.694 *r* = 0.141 *p* = 0.442 *r* = 0.466* *p* = 0.007 *r* = 0.076 *p* = 0.678 *r* = 0.079 *p* = 0.667	*r* = 0.298 *p* = 0.097 *r* = 0.069 *p* = 0.708 *r* = 0.445* *p* = 0.011 *r* = 0.501* *p* = 0.003 *r* = 0.315 *p* = 0.079 *r* = 0.274 *p* = 0.129	*r* = 0.390* *p* = 0.049 *r* = 0.594* *p* = 0.004 *r* = 0.458* *p* = 0.019 *r* = 0.481* *p* = 0.023 *r* = 0.520* *p* = 0.006 *r* = 0.443* *p* = 0.023

*Statistically significant (*p* < 0.05)

**Statistically significant at (*p* < 0.0006), Bonferroni-adjusted *p*-value

Higher percentages of CD4 and CD8 T cells double positive for PD-1, TIM-3, and/or TIGIT correlated with lower CD4 T cell counts, lower CD4 CD8 T cell ratios, and higher percentages of activated CD8 T cells. Higher percentages of CD8 T cells double positive for PD-1 and TIM-3, as well as with CD4 T cells double positive for PD-1 and TIGIT, correlated with lower nadir CD4 T cell counts. Higher percentages of CD4 T cells double positive for PD-1 and TIM-3 correlated with higher percentages of senescent CD4 T cells. Higher percentages of CD4 T cells double positive for PD-1 and TIM-3 and CD8 T cells double positive for TIM-3 and TIGIT correlated with higher percentages of senescent CD8 T cells.

### Relationship of NCR-expressing T cells and CD16^+^ monocyte subpopulations

Proportions of monocyte subpopulations were evaluated by flow-cytometry based on CD14 and CD16 expression. Correlations between single and dual NCR-expressing CD8 and CD4 T cells and monocyte subpopulation counts are shown in Table 4. Higher frequencies of CD4 T cells single positive for TIM-3 correlated with higher counts of intermediate (CD14 + + CD16+) monocytes. Higher percentages of

**Table 4:** Pearson correlations between NCR-expressing CD4 and CD8 T cells and monocyte subpopulations.

	Classical Monocytes	Intermediate Monocytes	Non-classical Monocytes
*Single positive*:			
CD8 + TIGIT+ CD8 + PD-1+ CD8 + TIM-3+ CD4 + TIGIT+ CD4 + PD-1+ CD4 + TIM-3+	*r*=-0.167 *p* = 0.415 *r*=-0.106 *p* = 0.606 *r*=-0.140 *p* = 0.496 *r* = 0.332 *p* = 0.098 *r* = 0.255 *p* = 0.208 *r* = 0.047 *p* = 0.818	*r*=-0.003 *p* = 0.987 *r* = 0.157 *p* = 0.443 *r* = 0.161 *p* = 0.433 *r* = 0.226 *p* = 0.267 *r* = 0.218 *p* = 0.285 *r* = 0.480* *p* = 0.013	*r* = 0.239 *p* = 0.239 *r* = 0.417* *p* = 0.034 *r* = 0.224 *p* = 0.271 *r* = 0.246 *p* = 0.225 *r* = 0.397* *p* = 0.045 *r* = 0.130 *p* = 0.525
*Dual positive*:			
CD8 + PD-1 + TIM-3+ CD8 + PD-1 + TIGIT+ CD8 + TIM-3 + TIGIT+ CD4 + PD-1 + TIM-3+ CD4 + PD-1 + TIGIT+ CD4 + TIM-3 + TIGIT+	*r*=-0.211 *p* = 0.302 *r*=-0.262 *p* = 0.197 *r*=-0.366 *p* = 0.066 *r* = 0.192 *p =* 0.384 *r* = 0.245 *p* = 0.227 *r* = 0.187 *p* = 0.359	*r* = 0.290 *p* = 0.151 *r* = 0.023 *p* = 0.911 *r* = 0.176 *p* = 0.391 *r* = 0.425* *p* = 0.031 *r* = 0.184 *p* = 0.368 *r* = 0.453* *p* = 0.020	*r* = 0.351 *p* = 0.079 *r* = 0.320 *p* = 0.111 *r* = 0.224 *p* = 0.271 *r* = 0.465* *p* = 0.017 *r* = 0.299 *p* = 0.138 *r* = 0.148 *p* = 0.469

*Statistically significant (*p* < 0.05)

both CD4 and CD8 T cells single positive for PD-1 correlated with higher counts of non-classical (CD14+/lowCD16++) monocytes.

Higher frequencies of CD4 T cells double positive for PD-1 and TIM-3 correlated with higher counts of both intermediate and non-classical monocytes. Higher frequencies of CD4 T cells double positive for TIM-3 and TIGIT correlated with higher counts of intermediate monocytes. No significant associations were found with activated and senescent T cells with monocyte subpopulations.

### Relationship of NCR-expressing CD4 T cells and NP performance

Significant correlations with NP test scores were found for NCR double positive CD4 T cells. No relationships with NP performance were observed with any NCR markers on CD8 T cells. Higher frequencies of CD4 T cells double positive for TIM-3 and PD-1 correlated with lower executive function (*r*=-0.427, *p* = 0.013) and working memory (*r*=-0.352, *p* = 0.045). CD4 T cells double positive for TIM-3 and TIGIT correlated with slower psychomotor speed (*r*=-0.350, *p* = 0.050). In multivariable linear regression models (Table 5) controlling for nadir CD4 count, higher frequencies of CD4 T cells double positive for PD-1 and TIM-3 remained associated with lower executive function (*p* < 0.001) and working memory (*p =* 0.009), with an association trend with overall NP performance (NPZ-Global; *p* = 0.056). In addition, higher frequencies of CD4 T cells double positive for TIM-3 and TIGIT remained associated with slower psychomotor speed (*p* = 0.018).

**Table 5:** Multivariable linear regression models between exhausted (PD-1^+^, TIM-3^+^, and/or TIGIT^+^) CD4 T cells and neuropsychological performance z-scores while controlling for nadir CD4 count.

Neurocognitive Domain (Dependent variable)	Predictor Variables	Unstandardized Coefficients		β value	*p-*value	95% C.I. for B-value	
		*B-Value*	*Std. error*			*Lower*	*Upper*
Executive Function	PD-1 + TIM-3 + CD4 T Cells	-0.235	0.057	-0.701	< 0.001*	-0.352	-0.119
	Nadir CD4 T Cell Counts	-0.003	0.001	-0.405	0.024	-0.006	0
Working Memory	PD-1 + TIM-3 + CD4 T Cells	-0.107	0.038	-0.532	0.009*	-0.184	-0.029
	Nadir CD4 T Cell Counts	-0.001	0.001	-0.185	0.338	-0.003	0.001
Global-14	PD-1 + TIM-3 + CD4 T Cells	-0.083	0.041	-0.423	0.056	-0.168	0.002
	Nadir CD4 T Cell Counts	-0.001	0.001	-0.31	0.153	-0.003	0.001
Psychomotor Speed	PD-1 + TIM-3 + CD4 T Cells	-0.147	0.059	-0.441	0.018*	-0.267	-0.027
	Nadir CD4 T Cell Counts	-0.259	0.171	-0.268	0.14	-0.608	0.09

*Statistically significant (*p* < 0.05)

### Relationship of T cells and regional brain volumes

In multivariable linear regression models (Table 6) controlling for age, nadir CD4 T cell count and intracranial volume, only CD8 T cells single and double positive for PD-1, TIM-3, and/or TIGIT were associated with regional brain volumes. Interestingly, no volumetric associations were found for NCRs on CD4 T cells. Higher frequencies of CD8 T cells single positive for PD-1 were associated with smaller volumes of the putamen, nucleus accumbens, cerebellar cortex, and subcortical gray matter. Higher frequencies of CD8 T cells double positive for PD-1 and TIM-3 were associated with smaller putamen and nucleus accumbens. Higher frequencies of CD8 T cells double positive for PD-1 and TIGIT were associated with only smaller volumes of the nucleus accumbens.

**Table 6:** Multivariable linear regression models between exhausted (PD-1^+^, TIM-3^+^, and/or TIGIT^+^) CD8 T cells and regional brain volumes.^†^

Brain Region Volume (mmm^3)	Predictor Variable	Unstandardized Coefficients		β value	*p*-value	95% C.I. for B-value	
		B-Value	Std. error			Lower	Upper
Putamen	PD-1 + CD8 T Cells	-49.015	15.696	-0.522	0.005*	-81.409	-16.621
	PD-1 + TIM-3 + CD8 T Cells	-161.61	49.048	-0.533	0.003*	-262.839	-60.381
	PD-1 + TIGIT + CD8 T Cells	-56.62	19.626	-0.488	0.008*	-97.124	-16.117
Nucleus Accumbens	PD-1 + CD8 T Cells	-6.608	2.714	-0.426	0.023*	-12.208	-1.007
	PD-1 + TIM-3 + CD8 T Cells	-18.595	8.843	-0.371	0.046*	-36.846	-0.344
Cerebellar Cortex	PD-1 + CD8 T Cells	-281.696	131.16	-0.389	0.042*	-552.397	-10.995
Subcortical Gray Matter	PD-1 + CD8 T Cells	-412.515	186.613	-0.368	0.037*	-797.664	-27.365

^†^Adjusted for age, nadir CD4 T cell count, and intracranial volume

*Statistically significant (*p* < 0.05)

### Correlations between monocyte subpopulations, NP performance, and brain volumes

Higher frequency of total monocyte correlated with lower psychomotor speed (*r* = -0.347, *p* = 0.04), while higher frequency of non-classical monocyte correlated with lower executive function (*r* = − 0.424, *p* = 0.022) and is shown in Supplemental Table 1. Higher frequencies of non-classical monocyte correlated with lower putamen volume (*r* = -0.378, *p* = 0.048), while higher total non-classical monocyte count correlated with lower cerebellar cortical (*r* = -0.430, *p* = 0.022) and lower putamen volumes (*r*= -0.385, *p* = 0.043) as shown in Supplemental Table 1. When adjusted for nadir CD4 count, only the following associations remained significant by multivariable linear regression: higher frequency of total monocyte with lower psychomotor speed (β = -0.367, *p* = 0.048) and higher frequency of non-classical monocyte with lower executive function (β = -0.466, *p* = 0.019) as shown in Supplemental Table 2.

## Discussion

Our study describes the novel relationship between dual expressing PD-1/TIM-3 CD4 and CD8 T cells with neurocognitive impairment and brain atrophy, respectively, and highlights NCR-expressing T cells as a potential novel target that may reverse cognitive impairment in HIV among PLWH on ART. In addition, our data suggests that a close relationship exists between NCR-expressing T cells and circulating monocyte subpopulations in the context of HIV.

As expected, our study found that higher frequencies of CD4 and CD8 T cells single and double positive for PD-1, TIM-3, and/or TIGIT supports previous findings showing the relationship between lower clinical measures of immune function, such as lower current CD4 count, CD4/CD8 T cell ratio, and nadir CD4 T cell count and HIV cognitive impairment(Fenwick et al. 2019). Importantly, parameters of dysregulated immune function, particularly the CD4/CD8 T cell ratio, have been linked to non-AIDS comorbidities, including subclinical atherosclerosis, lower renal function, muscle wasting, and to worsening cognitive function (Bernal Morell et al. 2016; Fenwick et al. 2019; Grauer et al. 2015; Serrano-Villar et al. 2014a, b) and that the exhausted T cell population may serve as a more accurate functional marker of HIV-associated comorbidities beyond the central nervous system.

Poorer executive function, working memory performance, decreased psychomotor speed, and poor everyday functioning have been identified in PLWH despite potent ART (Heaton et al. 2011; Koffler et al. 2018). Our study findings relating NCR-expressing CD4 T cell subpopulations to these subdomains suggests that they directly or indirectly affect cognitive impairment. By contrast, NCR expression on CD8 T cells are associated with smaller volumes of the putamen, nucleus accumbens, cerebellar cortex, and subcortical gray matter; areas known to be impacted by HIV despite ART(Ances and Hammoud 2014; Holt et al. 2012; Kallianpur et al. 2013, 2020). Grey matter volumes in cortical and subcortical regions have also been used to classify those with HAND with a high sensitivity and specificity further supporting an association between regional brain volumes and NP functioning in PLWH(Schantell et al. 2021). Studies have shown that brain regions responsible for executive functioning predominantly involves the prefrontal cortex, basal ganglia and thalamus(Royall et al. 2002). Working memory, on the other hand, primarily involves the fronto-parietal brain region and, more recently, subcortical regions have previously reported correlations between frailty characteristics and reduced volume in HIV(Chai et al. 2018; Kallianpur et al. 2016b). In the current study, we only identified one weakly positive correlation between cerebellar cortex ICV and executive functioning, but did not find any correlation between other brain volumes and NP z-scores (Supplemental Table 2). This was likely due to the small sample size of our cohort.

Review of the literature has revealed scant studies on the role of NCR-expressing T cells in the pathogenesis of HAND. Among PLWH with mild or severe neurocognitive alterations, one study has found increased PD-1 expression on memory CD4 + T cells, with the highest levels on effector memory T cells (Grauer et al. 2015). Interestingly, a recent mouse model tauopathy dementia showed that PD-1/PD-L1 checkpoint blockade led to increased immunomodulatory monocyte-derived macrophages and improved cognitive performance(Rosenzweig et al. 2019).

The relationships linking NCR-expressing CD4 T cells with NP performance and NCR-expressing CD8 T cells with brain volumes found in our study remain an enigma. This discordant relationship may be secondary to differences in the key function of these cells– i.e., CD4 T cells are more involved in pro-inflammatory cytokine production which may mediate neuropsychological dysfunction without cell loss, whereas CD8 T cell’s cytotoxic function may mediate neuronal loss resulting in structural changes (Subra and Trautmann 2019).

We found some correlations between NCR-expressing T cells and various monocyte subsets (Table 4). Ramello et al. reported that autologous monocytes co-cultured with CD4 or CD8 tumor-induced senescent (TIS)-T cells result in higher production of pro-inflammatory cytokines from monocyte/macrophages including TNF-α, IL-1β, and IL-6 (Ramello et al. 2014). It is worth noting that cell-to-cell interaction was required for the modulation of the monocyte/macrophages and that this was mediated by TIM-3 and CD40L (Ramello et al. 2014). It is possible that this interaction may be bi-directional as it is also known that monocyte/macrophages are effective antigen-presenting cells capable of activating both CD4 and CD8 T cells to effector phenotypes through the expression of antigen via HLA surface proteins(Ances and Hammoud 2014).

Significant correlations were also noted between monocytes, neurocognitive domains, and brain volumes. On multivariable linear regression adjusted for CD4 count, higher frequency of total monocyte was associated with lower psychomotor speed, while higher frequency of non-classical monocyte was associated with lower executive function. Similar to peripheral T cell exhaustion, peripheral monocytes likely play a role in CNS function since these cells have the ability to cross the blood-brain barrier. Previous studies have reported similar associations between monocytes and brain function (Clifford 2017; Fischer-Smith et al. 2001; Kusao et al. 2012; Ndhlovu et al. 2015; Shikuma et al. 2014; Veenstra et al. 2019; Williams et al. 2014).

This study has several limitations, including its cross-sectional nature, small sample size, and lack of an HIV-seronegative control for monocyte and T-cell NCR experiments. This study was not designed to establish the diagnosis of HAND. We performed several statistical analyses between variables. Due to sample size limitations and the exploratory nature of this manuscript, we did not plan on applying multiplicity corrections to our analyses. Additionally, our experiments focused on PBMCs and may not necessarily reflect immune dysregulation within the CNS, as we did not perform cerebrospinal fluid analyses. The strengths of this study, however, include the availability of NP performance and MRI data in an otherwise immunologically well-characterized cohort of HIV infected individuals on ART. Future directions include the use of a larger cohort to strengthen the statistical power, including monocyte and T-cell analyses among HIV-seronegative controls, and repeating the NP assessments in the context of newer ‘test and treat’ strategies using integrase-based ART regimens.

In conclusion, we report that dual expressing PD-1/TIM-3 CD4 and CD8 T cells are associated with neurocognitive impairment and brain atrophy, respectively. Results from this study highlight a potential novel interaction of NCR-expressing T cells with circulating blood monocytes, and a possible role of the pro-inflammatory consequence of this interaction on the development of HAND and brain atrophy in PLWH on ART. Novel immunotherapy targeting PD-1 and TIM-3 may be considered as treatment modalities for HAND.

## Electronic supplementary material

Below is the link to the electronic supplementary material.

Supplementary Material 1

## Data Availability

All supporting data for this study are available from the corresponding author upon reasonable request.
