# Supplementary material for: Dynamics of peripheral T cell exhaustion and monocyte subpopulations in neurocognitive impairment and brain atrophy in chronic HIV infection
Source: J Neurovirol. 2024 Jun 29;30(5-6):489–99. doi: 10.1007/s13365-024-01223-w (PMC11846764; doi:10.1007/s13365-024-01223-w)
Supplement: Supplementary file 1 — Supplementary Material 1 [file 13365_2024_1223_MOESM1_ESM.docx]

**Supplemental Table 1.** Pearson correlations between monocyte subpopulations, neuropsychological performance, and regional brain volumes**.**

|  | | Total Monocyte (%) | Classical Monocyte (%) | Nonclassical Monocyte (%) | Intermediate Monocyte (%) |
| --- | --- | --- | --- | --- | --- |
| Global-14 | r | -0.031 | 0.173 | -0.158 | 0.046 |
|  | p-value | 0.870 | 0.409 | 0.450 | 0.827 |
| Learning and Memory | r | 0.060 | 0.138 | 0.082 | 0.113 |
|  | p-value | 0.741 | 0.493 | 0.684 | 0.574 |
| Psychomotor Speed | r | **-.347^*^** | -0.020 | 0.029 | 0.198 |
|  | p-value | 0.044 | 0.921 | 0.882 | 0.313 |
| Executive Functioning | r | -0.058 | 0.355 | **-.424^*^** | -0.179 |
|  | p-value | 0.742 | 0.059 | 0.022 | 0.354 |
| Working Memory | r | -0.143 | 0.149 | -0.073 | -0.242 |
|  | p-value | 0.414 | 0.440 | 0.707 | 0.205 |
| Thalamus ICV | r | 0.119 | 0.046 | -0.154 | 0.007 |
|  | p-value | 0.503 | 0.818 | 0.432 | 0.971 |
| Caudate ICV | r | -0.009 | 0.177 | -0.246 | 0.085 |
|  | p-value | 0.960 | 0.369 | 0.207 | 0.669 |
| Putamen ICV | r | 0.124 | -0.045 | **-.378^*^** | 0.049 |
|  | p-value | 0.483 | 0.822 | 0.048 | 0.805 |
| Pallidum ICV | r | -0.105 | -0.040 | -0.029 | 0.088 |
|  | p-value | 0.554 | 0.839 | 0.883 | 0.657 |
| Hippocampus ICV | r | -0.034 | 0.053 | -0.243 | -0.084 |
|  | p-value | 0.847 | 0.789 | 0.213 | 0.670 |
| Amygdala ICV | r | -0.027 | 0.010 | -0.148 | -0.062 |
|  | p-value | 0.879 | 0.959 | 0.451 | 0.753 |
| Accumbens ICV | r | 0.202 | 0.099 | -0.306 | 0.117 |
|  | p-value | 0.251 | 0.616 | 0.113 | 0.554 |
| Total corpus callosum ICV | r | -0.139 | 0.068 | -0.087 | -0.097 |
|  | p-value | 0.433 | 0.732 | 0.659 | 0.623 |
| Cerebellar white matter ICV | r | 0.015 | -0.056 | 0.003 | -0.193 |
|  | p-value | 0.932 | 0.776 | 0.988 | 0.326 |
| Cerebellar cortex ICV | r | -0.061 | 0.085 | -0.342 | -0.060 |
|  | p-value | 0.731 | 0.666 | 0.075 | 0.762 |
| Cortical grey matter ICV | r | 0.041 | 0.037 | -0.213 | -0.016 |
|  | p-value | 0.818 | 0.850 | 0.278 | 0.938 |
| Cortical white matter ICV | r | -0.144 | 0.074 | -0.067 | 0.018 |
|  | p-value | 0.415 | 0.710 | 0.736 | 0.926 |
| Subcortical grey matter ICV | r | -0.020 | 0.051 | -0.302 | -0.066 |
|  | p-value | 0.910 | 0.796 | 0.119 | 0.738 |
| *Correlation is significant at the 0.05 level (2-tailed). | | | | | |
| **Correlation is significant at the 0.01 level (2-tailed).  Abbreviations: ICV, Intracranial Volume; r: Pearson’s correlation coefficient | | | | | |

**Supplemental Table 2.** Pearson Correlations between neuropsychological performance and regional brain volumes**.**

|  |  | Global NPZ Score | Learning and Memory NPZ Score | Psychomotor NPZ Score | Executive Functioning NPZ Score | Working Memory NPZ Score |
| --- | --- | --- | --- | --- | --- | --- |
| Thalamus ICV | r | 0.287 | 0.155 | 0.154 | 0.247 | 0.262 |
|  | p-value | 0.124 | 0.396 | 0.392 | 0.159 | 0.134 |
| Caudate ICV | r | 0.028 | 0.018 | 0.087 | 0.060 | -0.017 |
|  | p-value | 0.884 | 0.924 | 0.629 | 0.738 | 0.923 |
| Putamen ICV | r | 0.114 | -0.030 | 0.045 | 0.096 | 0.048 |
|  | p-value | 0.549 | 0.870 | 0.803 | 0.588 | 0.787 |
| Pallidum ICV | r | -0.168 | -0.076 | -0.034 | -0.061 | 0.095 |
|  | p-value | 0.375 | 0.681 | 0.849 | 0.730 | 0.592 |
| Hippocampus ICV | r | 0.079 | 0.105 | -0.064 | 0.072 | 0.170 |
|  | p-value | 0.677 | 0.569 | 0.723 | 0.686 | 0.338 |
| Amygdala ICV | r | -0.242 | -0.236 | -0.203 | -0.088 | 0.095 |
|  | p-value | 0.197 | 0.193 | 0.257 | 0.621 | 0.593 |
| Accumbens ICV | r | 0.217 | 0.123 | 0.038 | 0.079 | -0.037 |
|  | p-value | 0.250 | 0.503 | 0.832 | 0.658 | 0.834 |
| Total corpus callosum ICV | r | 0.048 | 0.097 | -0.004 | 0.073 | 0.176 |
|  | p-value | 0.801 | 0.597 | 0.982 | 0.683 | 0.319 |
| Cerebellar white matter ICV | r | 0.047 | -0.126 | 0.035 | 0.124 | 0.167 |
|  | p-value | 0.806 | 0.493 | 0.846 | 0.486 | 0.346 |
| Cerebellar cortex ICV | r | 0.316 | 0.114 | 0.173 | **.362^*^** | 0.086 |
|  | p-value | 0.089 | 0.535 | 0.335 | 0.036 | 0.629 |
| Cortical grey matter ICV | r | 0.123 | 0.170 | 0.041 | 0.049 | 0.112 |
|  | p-value | 0.516 | 0.353 | 0.822 | 0.783 | 0.529 |
| Cortical white matter ICV | r | 0.054 | 0.001 | 0.050 | 0.088 | 0.263 |
|  | p-value | 0.777 | 0.994 | 0.781 | 0.622 | 0.132 |
| Subcortical grey matter ICV | r | 0.240 | 0.089 | 0.116 | 0.277 | 0.141 |
|  | p-value | 0.201 | 0.630 | 0.521 | 0.112 | 0.428 |
| *Correlation is significant at the 0.05 level (2-tailed). | | | | | | |
| **Correlation is significant at the 0.01 level (2-tailed).  Abbreviations: ICV, Intracranial Volume; r: Pearson’s correlation coefficient | | | | | | |

**Supplemental Table 3.** Multivariable linear regression between monocyte subpopulations, neuropsychological performance, and regional brain volumes adjusted for nadir CD4 T cell counts**.**

|  |  | Unstandardized Coefficients | | Standardized Coefficients | | 95% C.I. for B-Value | |
| --- | --- | --- | --- | --- | --- | --- | --- |
|  |  | *B-Value* | *Std. Error* | Beta | p-value | *Lower* | *Upper* |
| Global-14 | Total Monocyte (%) | -0.017 | 0.048 | -0.069 | 0.729 | -0.115 | 0.081 |
|  | Nadir CD4 T Cell Counts | -0.001 | 0.001 | -0.137 | 0.495 | -0.002 | 0.001 |
| Learning and  Memory | Total Monocyte (%) | 0.017 | 0.078 | 0.043 | 0.826 | -0.143 | 0.178 |
|  | Nadir CD4 T Cell Counts | -0.001 | 0.002 | -0.083 | 0.669 | -0.004 | 0.002 |
| Psychomotor Speed | Total Monocyte (%) | -0.1 | 0.048 | -0.367 | 0.048 | -0.198 | -0.001 |
|  | Nadir CD4 T Cell Counts | 0 | 0.001 | -0.065 | 0.718 | -0.002 | 0.002 |
| Executive Functioning | Total Monocyte (%) | -0.024 | 0.071 | -0.062 | 0.739 | -0.168 | 0.121 |
|  | Nadir CD4 T Cell Counts | 8.7x10^-5^ | 0.001 | 0.012 | 0.95 | -0.003 | 0.003 |
| Working Memory | Total Monocyte (%) | -0.035 | 0.05 | -0.126 | 0.492 | -0.136 | 0.067 |
|  | Nadir CD4 T Cell Counts | 0.001 | 0.001 | 0.113 | 0.539 | -0.001 | 0.003 |
| Thalamus ICV | Total Monocyte (%) | 3.58x10^-5^ | 0 | 0.081 | 0.662 | 0 | 0 |
|  | Nadir CD4 T Cell Counts | -1.36x10^-6^ | 0 | -0.166 | 0.37 | 0 | 0 |
| Caudate ICV | Total Monocyte (%) | 1.46x10^-6^ | 0 | 0.006 | 0.976 | 0 | 0 |
|  | Nadir CD4 T Cell Counts | 4.91x10^-7^ | 0 | 0.102 | 0.587 | 0 | 0 |
| Putamen ICV | Total Monocyte (%) | 7.03x10^-5^ | 0 | 0.161 | 0.386 | 0 | 0 |
|  | Nadir CD4 T Cell Counts | 1.23x10^-6^ | 0 | 0.153 | 0.409 | 0 | 0 |
| Pallidum ICV | Total Monocyte (%) | -1.16x10^-5^ | 0 | -0.082 | 0.659 | 0 | 0 |
|  | Nadir CD4 T Cell Counts | 2.37x10^-7^ | 0 | 0.091 | 0.625 | 0 | 0 |
| Hippocampus ICV | Total Monocyte (%) | -1.51x10^-5^ | 0 | -0.047 | 0.801 | 0 | 0 |
|  | Nadir CD4 T Cell Counts | -3.12x10^-7^ | 0 | -0.053 | 0.777 | 0 | 0 |
| Amygdala ICV | Total Monocyte (%) | -5.16x10^-6^ | 0 | -0.03 | 0.874 | 0 | 0 |
|  | Nadir CD4 T Cell Counts | -7.74x10^-9^ | 0 | -0.002 | 0.99 | 0 | 0 |
| Accumbens ICV | Total Monocyte (%) | 1.40x10^-5^ | 0 | 0.233 | 0.207 | 0 | 0 |
|  | Nadir CD4 T Cell Counts | 1.32x10^-7^ | 0 | 0.119 | 0.514 | 0 | 0 |
| Total corpus callosum ICV | Total Monocyte (%) | -2.36x10^-5^ | 0 | -0.149 | 0.425 | 0 | 0 |
|  | Nadir CD4 T Cell Counts | -2.84x10^-7^ | 0 | -0.097 | 0.601 | 0 | 0 |
| Cerebellar white matter ICV | Total Monocyte (%) | -4.53x10^-5^ | 0 | -0.031 | 0.866 | -0.001 | 0 |
|  | Nadir CD4 T Cell Counts | -4.61x10^-6^ | 0 | -0.173 | 0.354 | 0 | 0 |
| Cerebellar cortex ICV | Total Monocyte (%) | 0 | 0.001 | -0.063 | 0.735 | -0.001 | 0.001 |
|  | Nadir CD4 T Cell Counts | -5.38x10^-8^ | 0 | -0.001 | 0.996 | 0 | 0 |
| Cortical grey matter ICV | Total Monocyte (%) | 0 | 0.002 | 0.032 | 0.862 | -0.005 | 0.005 |
|  | Nadir CD4 T Cell Counts | -2.17x10^-5^ | 0 | -0.089 | 0.634 | 0 | 0 |
| Cortical white matter ICV | Total Monocyte (%) | -0.003 | 0.003 | -0.183 | 0.319 | -0.008 | 0.003 |
|  | Nadir CD4 T Cell Counts | -4.96x10^-5^ | 0 | -0.186 | 0.312 | 0 | 0 |
| Subcortical grey matter ICV | Total Monocyte (%) | 0 | 0.001 | -0.024 | 0.898 | -0.002 | 0.002 |
|  | Nadir CD4 T Cell Counts | -4.62x10^-7^ | 0 | -0.005 | 0.98 | 0 | 0 |
| Global-14 | Classical Monocyte (%) | 0.923 | 1.164 | 0.17 | 0.437 | -1.497 | 3.344 |
|  | Nadir CD4 T Cell Counts | 0 | 0.001 | -0.078 | 0.72 | -0.003 | 0.002 |
| Learning and  Memory | Classical Monocyte (%) | 1.28 | 1.767 | 0.148 | 0.476 | -2.376 | 4.936 |
|  | Nadir CD4 T Cell Counts | -0.001 | 0.002 | -0.122 | 0.558 | -0.005 | 0.003 |
| Psychomotor Speed | Classical Monocyte (%) | -0.145 | 1.361 | -0.022 | 0.916 | -2.953 | 2.664 |
|  | Nadir CD4 T Cell Counts | -5.60x10-5 | 0.001 | -0.008 | 0.969 | -0.003 | 0.003 |
| Executive Functioning | Classical Monocyte (%) | 3.459 | 1.832 | 0.353 | 0.071 | -0.314 | 7.233 |
|  | Nadir CD4 T Cell Counts | 0 | 0.002 | -0.031 | 0.87 | -0.004 | 0.004 |
| Working Memory | Classical Monocyte (%) | 0.874 | 1.202 | 0.144 | 0.474 | -1.601 | 3.349 |
|  | Nadir CD4 T Cell Counts | 0 | 0.001 | -0.036 | 0.857 | -0.003 | 0.002 |
| Thalamus ICV | Classical Monocyte (%) | 0 | 0.002 | 0.045 | 0.829 | -0.004 | 0.005 |
|  | Nadir CD4 T Cell Counts | -6.80x10^-7^ | 0 | -0.064 | 0.758 | 0 | 0 |
| Caudate ICV | Classical Monocyte (%) | 0.001 | 0.001 | 0.161 | 0.418 | -0.002 | 0.004 |
|  | Nadir CD4 T Cell Counts | 1.52x10^-6^ | 0 | 0.235 | 0.242 | 0 | 0 |
| Putamen ICV | Classical Monocyte (%) | -0.001 | 0.002 | -0.057 | 0.772 | -0.005 | 0.004 |
|  | Nadir CD4 T Cell Counts | 3.44x10^-6^ | 0 | 0.321 | 0.111 | 0 | 0 |
| Pallidum ICV | Classical Monocyte (%) | 0 | 0.001 | -0.049 | 0.807 | -0.002 | 0.001 |
|  | Nadir CD4 T Cell Counts | 8.63x10^-7^ | 0 | 0.247 | 0.223 | 0 | 0 |
| Hippocampus ICV | Classical Monocyte (%) | 0 | 0.001 | 0.044 | 0.828 | -0.003 | 0.003 |
|  | Nadir CD4 T Cell Counts | 1.12x10^-6^ | 0 | 0.15 | 0.466 | 0 | 0 |
| Amygdala ICV | Classical Monocyte (%) | 3.32x10^-6^ | 0.001 | 0.001 | 0.997 | -0.002 | 0.002 |
|  | Nadir CD4 T Cell Counts | 6.55x10^-7^ | 0 | 0.16 | 0.435 | 0 | 0 |
| Accumbens ICV | Classical Monocyte (%) | 0 | 0 | 0.087 | 0.651 | 0 | 0.001 |
|  | Nadir CD4 T Cell Counts | 4.98x10^-7^ | 0 | 0.353 | 0.075 | 0 | 0 |
| Total corpus callosum ICV | Classical Monocyte (%) | 0 | 0.001 | 0.077 | 0.709 | -0.001 | 0.002 |
|  | Nadir CD4 T Cell Counts | 1.44x10^-7^ | 0 | 0.037 | 0.857 | 0 | 0 |
| Cerebellar white matter ICV | Classical Monocyte (%) | -0.002 | 0.007 | -0.065 | 0.754 | -0.016 | 0.012 |
|  | Nadir CD4 T Cell Counts | -1.56x10^-6^ | 0 | -0.045 | 0.827 | 0 | 0 |
| Cerebellar cortex ICV | Classical Monocyte (%) | 0.005 | 0.014 | 0.074 | 0.713 | -0.024 | 0.035 |
|  | Nadir CD4 T Cell Counts | 1.44x10^-5^ | 0 | 0.195 | 0.34 | 0 | 0 |
| Cortical grey matter ICV | Classical Monocyte (%) | 0.014 | 0.063 | 0.046 | 0.824 | -0.116 | 0.144 |
|  | Nadir CD4 T Cell Counts | 4.14x10^-7^ | 0 | 0.001 | 0.995 | 0 | 0 |
| Cortical white matter ICV | Classical Monocyte (%) | 0.027 | 0.069 | 0.079 | 0.701 | -0.116 | 0.17 |
|  | Nadir CD4 T Cell Counts | -3.98x10^-5^ | 0 | -0.113 | 0.582 | 0 | 0 |
| Subcortical grey matter ICV | Classical Monocyte (%) | 0.005 | 0.025 | 0.04 | 0.844 | -0.046 | 0.056 |
|  | Nadir CD4 T Cell Counts | 2.44x10^-5^ | 0 | 0.193 | 0.346 | 0 | 0 |
| Global-14 | Nonclassical Monocyte (%) | -2.61 | 3.047 | -0.189 | 0.401 | -8.947 | 3.728 |
|  | Nadir CD4 T Cell Counts | -0.001 | 0.001 | -0.128 | 0.57 | -0.003 | 0.002 |
| Learning and  Memory | Nonclassical Monocyte (%) | 0.994 | 4.684 | 0.046 | 0.834 | -8.696 | 10.683 |
|  | Nadir CD4 T Cell Counts | -0.001 | 0.002 | -0.105 | 0.631 | -0.005 | 0.003 |
| Psychomotor Speed | Nonclassical Monocyte (%) | 0.502 | 3.493 | 0.03 | 0.887 | -6.707 | 7.711 |
|  | Nadir CD4 T Cell Counts | -6.92x10^-6^ | 0.001 | -0.001 | 0.996 | -0.003 | 0.003 |
| Executive Functioning | Nonclassical Monocyte (%) | -11.201 | 4.46 | -0.466 | 0.019 | -20.387 | -2.016 |
|  | Nadir CD4 T Cell Counts | -0.002 | 0.002 | -0.152 | 0.421 | -0.005 | 0.002 |
| Working Memory | Nonclassical Monocyte (%) | -1.257 | 3.084 | -0.084 | 0.687 | -7.609 | 5.095 |
|  | Nadir CD4 T Cell Counts | 0 | 0.001 | -0.057 | 0.786 | -0.003 | 0.002 |
| Thalamus ICV | Nonclassical Monocyte (%) | -0.005 | 0.005 | -0.191 | 0.375 | -0.016 | 0.006 |
|  | Nadir CD4 T Cell Counts | -1.31x10^-6^ | 0 | -0.122 | 0.569 | 0 | 0 |
| Caudate ICV | Nonclassical Monocyte (%) | -0.003 | 0.003 | -0.186 | 0.375 | -0.009 | 0.004 |
|  | Nadir CD4 T Cell Counts | 1.20x10^-6^ | 0 | 0.184 | 0.379 | 0 | 0 |
| Putamen ICV | Nonclassical Monocyte (%) | -0.008 | 0.005 | -0.315 | 0.117 | -0.018 | 0.002 |
|  | Nadir CD4 T Cell Counts | 2.33x10^-6^ | 0 | 0.217 | 0.274 | 0 | 0 |
| Pallidum ICV | Nonclassical Monocyte (%) | 0 | 0.002 | 0.05 | 0.811 | -0.003 | 0.004 |
|  | Nadir CD4 T Cell Counts | 9.10x10^-7^ | 0 | 0.261 | 0.223 | 0 | 0 |
| Hippocampus ICV | Nonclassical Monocyte (%) | -0.004 | 0.004 | -0.216 | 0.31 | -0.012 | 0.004 |
|  | Nadir CD4 T Cell Counts | 6.21x10^-7^ | 0 | 0.083 | 0.693 | 0 | 0 |
| Amygdala ICV | Nonclassical Monocyte (%) | -0.001 | 0.002 | -0.108 | 0.614 | -0.005 | 0.003 |
|  | Nadir CD4 T Cell Counts | 5.14x10^-7^ | 0 | 0.126 | 0.558 | 0 | 0 |
| Accumbens ICV | Nonclassical Monocyte (%) | -0.001 | 0.001 | -0.222 | 0.269 | -0.002 | 0.001 |
|  | Nadir CD4 T Cell Counts | 4.05x10^-7^ | 0 | 0.287 | 0.155 | 0 | 0 |
| Total corpus callosum ICV | Nonclassical Monocyte (%) | -0.001 | 0.002 | -0.091 | 0.674 | -0.005 | 0.003 |
|  | Nadir CD4 T Cell Counts | 4.74x10^-8^ | 0 | 0.012 | 0.955 | 0 | 0 |
| Cerebellar white matter ICV | Nonclassical Monocyte (%) | -0.001 | 0.018 | -0.007 | 0.975 | -0.037 | 0.036 |
|  | Nadir CD4 T Cell Counts | -1.76x10^-6^ | 0 | -0.051 | 0.815 | 0 | 0 |
| Cerebellar cortex ICV | Nonclassical Monocyte (%) | -0.055 | 0.036 | -0.31 | 0.137 | -0.129 | 0.019 |
|  | Nadir CD4 T Cell Counts | 7.40x10^-6^ | 0 | 0.1 | 0.625 | 0 | 0 |
| Cortical grey matter ICV | Nonclassical Monocyte (%) | -0.185 | 0.159 | -0.244 | 0.255 | -0.512 | 0.142 |
|  | Nadir CD4 T Cell Counts | -2.35x10^-5^ | 0 | -0.074 | 0.727 | 0 | 0 |
| Cortical white matter ICV | Nonclassical Monocyte (%) | -0.094 | 0.179 | -0.112 | 0.603 | -0.463 | 0.275 |
|  | Nadir CD4 T Cell Counts | -5.08x10^-5^ | 0 | -0.145 | 0.504 | 0 | 0 |
| Subcortical grey matter ICV | Nonclassical Monocyte (%) | -0.08 | 0.062 | -0.266 | 0.205 | -0.208 | 0.047 |
|  | Nadir CD4 T Cell Counts | 1.39x10^-5^ | 0 | 0.11 | 0.595 | 0 | 0 |
| Global-14 | Intermediate Monocyte (%) | 0.807 | 7.105 | 0.025 | 0.911 | -13.968 | 15.583 |
|  | Nadir CD4 T Cell Counts | 0 | 0.001 | -0.076 | 0.736 | -0.003 | 0.002 |
| Learning and  Memory | Intermediate Monocyte (%) | 7.137 | 10.221 | 0.144 | 0.492 | -14.006 | 28.28 |
|  | Nadir CD4 T Cell Counts | -0.001 | 0.002 | -0.106 | 0.611 | -0.005 | 0.003 |
| Psychomotor Speed | Intermediate Monocyte (%) | 7.518 | 7.845 | 0.193 | 0.347 | -8.674 | 23.709 |
|  | Nadir CD4 T Cell Counts | 8.07x10^-5^ | 0.001 | 0.012 | 0.955 | -0.003 | 0.003 |
| Executive Functioning | Intermediate Monocyte (%) | -12.93 | 11.114 | -0.227 | 0.256 | -35.819 | 9.959 |
|  | Nadir CD4 T Cell Counts | 0 | 0.002 | -0.044 | 0.822 | -0.004 | 0.004 |
| Working Memory | Intermediate Monocyte (%) | -11.286 | 6.705 | -0.32 | 0.105 | -25.095 | 2.524 |
|  | Nadir CD4 T Cell Counts | 0 | 0.001 | -0.059 | 0.758 | -0.003 | 0.002 |
| Thalamus ICV | Intermediate Monocyte (%) | -0.001 | 0.012 | -0.024 | 0.909 | -0.027 | 0.024 |
|  | Nadir CD4 T Cell Counts | -6.82x10^-7^ | 0 | -0.064 | 0.759 | 0 | 0 |
| Caudate ICV | Intermediate Monocyte (%) | 0.004 | 0.007 | 0.1 | 0.619 | -0.011 | 0.019 |
|  | Nadir CD4 T Cell Counts | 1.65x10^-6^ | 0 | 0.254 | 0.211 | 0 | 0 |
| Putamen ICV | Intermediate Monocyte (%) | 0.007 | 0.012 | 0.117 | 0.55 | -0.017 | 0.031 |
|  | Nadir CD4 T Cell Counts | 3.54x10^-6^ | 0 | 0.331 | 0.1 | 0 | 0 |
| Pallidum ICV | Intermediate Monocyte (%) | 0.003 | 0.004 | 0.147 | 0.462 | -0.005 | 0.011 |
|  | Nadir CD4 T Cell Counts | 9.11x10^-7^ | 0 | 0.261 | 0.197 | 0 | 0 |
| Hippocampus ICV | Intermediate Monocyte (%) | -0.003 | 0.008 | -0.073 | 0.72 | -0.021 | 0.014 |
|  | Nadir CD4 T Cell Counts | 1.08x10^-6^ | 0 | 0.144 | 0.484 | 0 | 0 |
| Amygdala ICV | Intermediate Monocyte (%) | -0.001 | 0.005 | -0.05 | 0.807 | -0.011 | 0.008 |
|  | Nadir CD4 T Cell Counts | 6.32x10^-7^ | 0 | 0.155 | 0.453 | 0 | 0 |
| Accumbens ICV | Intermediate Monocyte (%) | 0.002 | 0.001 | 0.196 | 0.305 | -0.002 | 0.005 |
|  | Nadir CD4 T Cell Counts | 5.35x10^-7^ | 0 | 0.38 | 0.054 | 0 | 0 |
| Total corpus callosum ICV | Intermediate Monocyte (%) | -0.001 | 0.004 | -0.048 | 0.816 | -0.01 | 0.008 |
|  | Nadir CD4 T Cell Counts | 1.40x10^-7^ | 0 | 0.036 | 0.862 | 0 | 0 |
| Cerebellar white matter ICV | Intermediate Monocyte (%) | -0.051 | 0.039 | -0.26 | 0.202 | -0.13 | 0.029 |
|  | Nadir CD4 T Cell Counts | -2.69x10^-6^ | 0 | -0.077 | 0.699 | 0 | 0 |
| Cerebellar cortex ICV | Intermediate Monocyte (%) | -0.017 | 0.084 | -0.041 | 0.84 | -0.19 | 0.156 |
|  | Nadir CD4 T Cell Counts | 1.44x10^-5^ | 0 | 0.194 | 0.344 | 0 | 0 |
| Cortical grey matter ICV | Intermediate Monocyte (%) | 0.045 | 0.365 | 0.026 | 0.902 | -0.708 | 0.799 |
|  | Nadir CD4 T Cell Counts | 2.11x10^-6^ | 0 | 0.007 | 0.974 | 0 | 0 |
| Cortical white matter ICV | Intermediate Monocyte (%) | 0.001 | 0.403 | 0 | 0.999 | -0.83 | 0.832 |
|  | Nadir CD4 T Cell Counts | -3.82x10^-5^ | 0 | -0.109 | 0.599 | 0 | 0 |
| Subcortical grey matter ICV | Intermediate Monocyte (%) | -0.037 | 0.143 | -0.052 | 0.799 | -0.331 | 0.258 |
|  | Nadir CD4 T Cell Counts | 2.39x10^-5^ | 0 | 0.189 | 0.357 | 0 | 0 |
| Abbreviations: ICV, Intracranial Volume; r: Pearson’s correlation coefficient | | | | | | | |
